# Detection of anti-HspX antibodies and HspX protein in patient sera for the identification of recent latent infection by *Mycobacterium tuberculosis*

**DOI:** 10.1371/journal.pone.0181714

**Published:** 2017-08-16

**Authors:** Jorge Castro-Garza, Paola García-Jacobo, Lydia G. Rivera-Morales, Frederick D. Quinn, James Barber, Russell Karls, Debra Haas, Shelly Helms, Tuhina Gupta, Henry Blumberg, Jane Tapia, Itza Luna-Cruz, Adrián Rendon, Javier Vargas-Villarreal, Lucio Vera-Cabrera, Cristina Rodríguez-Padilla

**Affiliations:** 1 Centro de Investigación Biomédica del Noreste, Instituto Mexicano del Seguro Social, Monterrey, Nuevo León, México; 2 Laboratorio de Inmunología y Virología, Facultad de Ciencias Biológicas, Universidad Autónoma de Nuevo León, San Nicolás de los Garza, Nuevo León, México; 3 Department of Infectious Diseases, University of Georgia, Athens, Georgia, United States of America; 4 Division of Infectious Diseases, Emory University School of Medicine, Atlanta, Georgia, United States of America; 5 Centro de Investigación, Prevención y Tratamiento de Infecciones Respiratorias (CIPTIR), Hospital Universitario, Universidad Autónoma de Nuevo León, Monterrey, Nuevo León, México; 6 Servicio de Dermatología, Hospital Universitario, Universidad Autónoma de Nuevo León, Monterrey, Nuevo León, México; Colorado State University, UNITED STATES

## Abstract

*Mycobacterium tuberculosis* is a pathogen causing tuberculosis (TB) a spectrum of disease including acute and asymptomatic latent stages. Identifying and treating latently-infected patients constitutes one of the most important impediments to TB control efforts. Those individuals can remain undiagnosed for decades serving as potential reservoirs for disease reactivation. Tests for the accurate diagnosis of latent infection currently are unavailable. HspX protein (α-crystallin), encoded by *Rv2031c* gene, is produced *in vitro* by *M*. *tuberculosis* during stationary growth phase and hypoxic or acidic culture conditions. In this study, using standard, and Luminex xMAP^®^ bead capture ELISA, respectively, we report on detection of anti-HspX IgG and IgM antibodies and HspX protein in sera from acute and latent TB patients. For the antibody screen, levels of IgG and IgM antibodies were similar between non-infected and active TB patients; however, individuals classified into the group with latent TB showed higher values of anti-HspX IgM (p = 0.003) compared to active TB patients. Using the bead capture antigen detection assay, HspX protein was detected in sera from 56.5% of putative latent cases (p< 0.050) compared to the background median with an average of 9,900 pg/ml and a range of 1,000 to 36,000 pg/ml. Thus, presence of anti-HspX IgM antibodies and HspX protein in sera may be markers of latent TB.

## Introduction

Tuberculosis (TB) is a bacterial disease primarily caused by infection with *Mycobacterium tuberculosis*. In 2014, an estimated 9.6 million people developed TB and 1.5 million died from the disease (including 400,000 deaths among HIV-positive individuals). In addition, approximately 480,000 multidrug resistant TB (MDR-TB) cases were reported [1]. Until a protective TB vaccine is developed, accurate diagnosis and effective treatment are the most useful tools available for controlling this disease.

Latent TB infection (LTBI) is defined as a state of persistent immune response to stimulation by *M*. *tuberculosis* antigens without evidence of clinically manifested active TB [2, 3]. Persons with LTBI have negative bacteriological tests and the presumptive diagnosis is based on a positive result of either a skin (tuberculin skin test, TST) or blood (interferon-gamma release assay, IGRA) test indicating an immune response to *M*. *tuberculosis* infection. There are treatment regimens that can reduce but not eliminate the risk of reactivation disease in individuals with LTBI, but such prophylactic therapy involves taking antibiotics for up to 9 months, and carries a small risk of serious, or even fatal, side-effects in these TST/IGRA-positive individuals only suspected of having LTBI. At present, a direct measurement tool for LTBI in humans is currently unavailable [2, 3], and the inability to identify the population of individuals with LTBI constitutes a major impediment to TB control efforts [4]. An Institute of Medicine report concluded that the development of new diagnostics that can distinguish the truly infected individuals from those who are exposed but self-cured/false positive will be the key to ultimately eliminating TB [5].

Proteins expressed under stress are ubiquitous in nature and they play an important role in helping bacterial cells to survive under extreme conditions. It is hypothesized that the primary location for latent *M*. *tuberculosis* bacilli is the granuloma, and within these structures, the bacteria are exposed to adverse conditions including hypoxic stress, low pH, hydrolytic enzymes, toxic fatty acids and reactive oxygen radicals. To survive, bacilli upregulate stress-response proteins including chaperones and transport proteins associated with pH control, metabolite movement, and lipid metabolism [6–9]. The *M*. *tuberculosis Rv2031c* gene encodes HspX (also known as α-crystallin, Acr, or the 16-kDa antigen) which is expressed during stationary growth, under hypoxia, and low pH, similar to conditions present within areas of some granulomas [10]. HspX may be an important element of *M*. *tuberculosis* replication control in the granuloma since its over-expression inhibits *M*. *tuberculosis* growth [11–13].

The use of a serodiagnostic test for TB was analyzed by Limongi et al. [14], who reported that specific anti-HspX IgA in pleural fluid samples was useful to discriminate between pleural effusion due to TB and other pulmonary disease. Similarly, detection of IgG, IgA, and IgM antibodies against the *M*. *tuberculosis*16-kDa antigen were evaluated by ELISA as a diagnostic tool in sera from TB patients and healthy subjects [15]. A combination of the three isotypes gave a sensitivity of 83% and specificity of 93% while precipitated circulating immune complexes of these antibodies improved the sensitivity to 97.5%. Interestingly, Steingart et al. [16] reported a commercial antibody detection test for the diagnosis of pulmonary TB that detects IgG against the *M*. *tuberculosis* 38-kDa and 16-kDa antigens, but does not include the detection of IgM.

Alternatively, two groups have reported that they are examining direct detection of *M*. *tuberculosis* antigens released into body fluids, however, those tests target abundant non-specific mycobacterial cell wall components: lipoarabinomannan (LAM) in urine [17, 18]. The relative reluctance of investigators to employ the detection of a low abundance secreted protein as a diagnostic is due to uncertainty as to what specific antigens are most appropriate, and the expectation that these factors may only be present in very low amounts in serum, especially in the case of LTBI.

As described, HspX is produced exclusively during periods of bacterial stress, including survival within granulomas [19]. In that study, naphthol red-tagged anti-HspX monoclonal antibody highlighted the presence of HspX protein on bacterial outer membranes within guinea pig early granulomas (3 weeks after infection) and later granulomas (10 weeks post-infection). Based on these and other unpublished studies, it was expected that this protein would be present in patient sera in picogram/ml to femtogram/ml concentrations; theoretically detectable amounts by ultra-sensitive detection systems. In this study, we report on successful detection of anti-HspX antibodies and HspX protein in LTBI patient sera, and statistically determine diagnostic potential.

## Materials and methods

### Ethics statement

The present study and the consent procedures were specifically approved by the Review Board of the National Research Commission of Instituto Mexicano del Seguro Social with approval number: R-2011-1906-41 in Mexico and The University of Georgia, Emory University and the DeKalb County, Georgia, and Board of Health Institutional Review Boards in United States of America.

### Study populations

#### Anti-HspX antibody detection ELISA

Serum samples of 98 adults were provided by the Center for Research, Prevention and Treatment of Respiratory Infections (CIPTIR) Hospital Universitario "Dr. José Eleuterio González", in Monterrey, Mexico. All of the individuals signed an informed consent before sampling. Patients were classified into four groups: Group 1: active TB, positive tuberculin test (PPD+,(induration greater than 10 mm), positive interferon-gamma release assay (QuantiFERON^®^, QFT+), positive smear test and positive microorganism culture (n = 28); Group 2: LTBI, PPD+, contact with active TB persons (COMBE+), with or without history of TB in the family (n = 30); Group 3: uninfected individuals, negative tuberculin test (PPD-) and absence of symptoms (n = 15); and Group 4: unknown status, inconsistent results between tests (n = 25).

#### Luminex xMAP^®^HspX protein detection ELISA

Sixty adults were enrolled at the DeKalb County (Georgia, USA) Board of Health Refugee Program/Refugee Clinic, and TB and LTBI clinics. Students entering the Emory University School of Medicine were a source of uninfected controls. All individuals signed an informed consent before sampling. However, unlike the anti-HspX antibody detection ELISA, patients were classified into only three groups: Group 1: active TB, positive tuberculin test (PPD+, induration greater than 10 mm) or positive interferon-gamma release assay (QuantiFERON^®^, QFT+), positive smear test and positive microorganism culture (n = 13); Group 2: LTBI, PPD+, contact with active TB persons (COMBE+), with or without history of TB in the family (n = 23); and Group 3: uninfected individuals, negative tuberculin test (PPD-) and absence of symptoms (n = 24).

All blood samples (10 ml) were collected by venipuncture, allowed to clot at room temperature (20–25°C) and then centrifuged according to the clinical and Laboratory Standards Institute (CLSI-Approved Standard-Procedures for the collection of Diagnostic Blood Specimens by Venipuncture, H3-A4, 1998). The serum was separated and frozen (-20°C) until analysis was performed.

### Construction of recombinant plasmid pET-23b(+)*hspX*

The sequence of *Rv2031c* (TB database: GB: AL123456) was used to design primers. The sequences for *Hin*dIII and *Xho*I cleavage sites were added to the 5′ end of forward PHspXF (**AAGCTT**ATGGCCACCACCCTTCCC) and reverse PHspXR (**CTCGAG**GTTGGTGGACCGGATCT) primers, respectively (bold letters correspond to restriction sites). The sequence encoding for *M*. *tuberculosis hspX* gene (*Rv2031c*) was PCR amplified from the *M*. *tuberculosis* H37Rv genome using a hot-start enzyme (Platinum^®^Taq DNA Polymerase) and subcloned into a C-terminal His-tag pET23b(+) vector (Novagen) using *Hind*III and *Xho*I restrictions sites following manufacturer instructions. Competent *Escherichia coli* BL21 (DE3)pLysS (Promega) was transformed with the plasmid containing the *hspX* gene. Recombinant bacteria were grown in LB broth for 1.5 hr at 37°C with shaking and subsequently 100 μl of culture were plated on LB agar (Invitogen, Carlsbad, CA) with ampicillin (100 μg/ml) (Amp^100^) and chloramphenicol (50 μg/ml) (Cam^50^). Subsequently, the pET-23b(+)*hspX* vector was purified using Wizard^®^Plus SV Minipreps ADN Purification System (Promega Corporation, Madison, WI) with a subsequent sequence confirmation step.

### Production and purification of the recombinant HspX protein

Recombinant HspX protein was purified from BL21 (DE3) pLysS cells transformed with pET23b(+)*hspX*. Briefly, a recombinant clone was grown overnight in 3.5 ml of LB broth with Amp^100^ and Cam^50^ with shaking (200 rpm). Three milliliters of bacterial suspension were inoculated into 300 ml of fresh LB broth with Amp^100^ and Cam^50^ and incubated at 37°C with shaking (200 rpm) to OD_600_ = 0.5. The culture was IPTG-induced (final concentration of 1 mM) for 3 hr with shaking (200 rpm) at 37°C. Purified protein was obtained using affinity chromatography with a Ni+ binding column and dialysis. Each purification step was confirmed on a 14% SDS-PAGE. Final concentration of purified recombinant HspX was 0.66 mg/ml.

Native HspX protein, obtained from BEI Resources, has a predicted molecular mass of 16-kDa, while the rHspX has a predicted molecular mass of approximately 20-kDa due to the added upstream T7 tag and downstream 6-His tag.

### Detection of HspX recombinant protein by western blot

Purified protein (0.36 mg/ml) was resolved by 14% SDS-PAGE. Subsequently, proteins were transferred to a nitrocellulose membrane at 35V and 4°C overnight (Mini Trans-Blot Cell (Bio-Rad). The membrane was incubated with blocking buffer (PBS 1X, 5% skim milk, 0.1% Tween 20) overnight with orbital shaking (4°C, 150 rpm), and washed 5X with PBS-T 1X (PBS 1X, 0.1% Tween 20). The membrane was incubated with 50 ml of a 1:100 dilution of anti-HspX monoclonal antibody (clone IT-20 NR-13607, from BEI Resources) as primary antibody at 4°C with shaking (150 rpm) overnight. After 5X washes (10 min each) with 1X PBS-T, 50 ml of the polyclonal secondary antibody (1:1500 dilution) goat anti-mouse IgG conjugated with HRP (Abcam, Cambridge, UK) was added and incubated for 2 hr at 4°C followed by 5X washes with 1X PBS-T. Finally, protein was detected by chemiluminescence using the SuperSignal^®^West Pico Chemiluminiscent Substrate (Pierce Biotechnology, Rockford, IL) following manufacturer´s recommendations.

### Analysis of cross-reacting *M*. *tuberculosis* HspX homologs

An *in silico* analysis was performed to identify proteins homologous to *M*. *tuberculosis* HspX. The resulting protein sequences were compared using program DNASTAR to obtain the percentage of similarity and divergence with HspX (S1 and S2 Tables). Mycobacterial genomes selected for analysis were: *M*. *tuberculosis* H37Rv, *M*. *bovis* BCG, *M*. *bovis*, *M*. *scrofulaceum*, *M*. *kansasii*, *M*. *intracellulare*, *M*. *smegmatis*, *M*. *rhodesiae*, *M*. *fortuitum*, *M*. *vaccae*, *M*. *avium*, and *M*. *tuberculosis* Beijing genotype. Bacteria were grown on 30 ml Middlebrook 7H9 + OADC broth during 20 days at 37°C in sealed tubes to favor oxygen depletion conditions [8]; except *M*. *tuberculosis* Beijing, cultures were harvested after 7 days. Harvested cells were resuspended in 1 ml of TBS (100 mM, pH 7.4, 0.5% Triton X-100 and EDTA-free protease inhibitor cocktail (Boehringer Manheim, Germany) and lysed in a FastPrep^®^ instrument using 3 cycles of 45 sec at speed 5 with cooling on ice between runs. Cross-reaction was analyzed by western blot as described previously using 0.36 mg/ml of protein of each lysate.

### Detection of IgG and IgM antibodies against HspX protein in TB patient sera by ELISA

Microplates (96-well) coated with nickel (Thermo Scientific Nunc Immobilizer Nickel—Chelate, Waltham, MA) were pre-washed 3X (300 μl/well) with PBST (PBS + 0.05% Tween 20 and covered with 50 μl of 10 μg/ml purified recombinant protein HspX-His previously dissolved in KCl 0.01 M. Plates were incubated at 4°C with shaking (450 rpm) overnight. Plates then were washed 3X with 300 μl PBST and blocked with 300 μl of 5% skim milk for 2 hr, followed by 3 washes with PBST (300 μl ea.). Prepared plates were loaded with 100 μl per well of patient serum (1:50 dilution in1% skim milk) and incubated at 37°C for 90 min. After incubation, the plates were washed with PBST (3 x 300 μl) and 100 μl of secondary antibody-HRP (goat anti-human IgM at 1:10,000 or anti-human IgG at 1:30,000) (Sigma Aldrich) were added and incubated for 1 hr at 37°C. Plates were washed again with PBST (3 x 300 μl) and 100 μl of Sigma Fast OPD solution (Peroxidase substrate, Sigma Aldrich, St Louis, MO) was added to each well. After 30 min incubation, the reaction was stopped with 50 μl of 3N sulfuric acid. ELISA absorbance readings at 492 nm were taken using a microplate reader Synergy 2 (BioTek, Inc., Winooski, VT).

### Detection of HspX protein in TB patient sera by Luminex xMAP^®^bead capture ELISA

A collection of 30 mouse anti-*M*. *tuberculosis* HspX (Gene *Rv2031*) monoclonal antibodies, including IT-20 (NR-13607), IT-49 (NR-13814), and IT-50 (NR-13815) obtained from BEI Resources NIH NIAID, were screened for optimal sensitivity and specificity in a Luminex xMAP^®^/Magpix (Luminex corp, Austin, TX) bead-capture ELISA system [20, 21]. This system was used to further enhance detection sensitivity compared to standard ELISA. The selected antibody pair, IT-20 (IgG1κ) and PAC326 (IgG2aκ) (kindly provided by D. Bagarrozi, CDC), generated against rHspX protein, were found to produce strong reactivity signals with routine detection of native or rHspX in spiked sera in the 1–10 pg/ml range. Luminex provides kits of amine-reactive paramagnetic fluorescent microspheres magnetized beads and a flow cytometry assay detection device (Magpix) as an open platform upon which research groups can develop custom assays using their own reagents. Assay methods recommended by the manufacturer were employed. Carboxylated beads were activated with an xMAP Antibody Coupling Kit (Luminexcorp, Austin, TX) and covalently conjugated with anti-HspX antibody, clone IT-20, according to manufacturer’s instructions. Capture antibody-coated beads were reacted with samples or rHspX-spiked standards and subsequently incubated with anti-HspX reporter antibody, clone PAC326, which was previously biotinylated with an EZ-Link Micro NHS-PEO4-Biotinylation Kit (Pierce Biotechnology, Rockford, IL). Beads were then developed with PhycoLink^®^ Streptavidin-R-Phycoerythrin (ProZyme, Hayward, CA). Data were collected on a Magpix instrument using xPONENT v.4.2 (Luminexcorp) and analyzed with Milliplex^®^ Analyst software, v.5.1 (EMD Millipore, Billerica, MA).

### Statistical analysis

#### Anti-HspX antibody detection ELISA

Measures of central tendency and variability were calculated from the absorbance values of anti-HspX IgG and IgM. The normality test K2 D'Agostino-Pearson was used to assess data. Differences between groups were analyzed by the Kruskal-Wallis and Dunn’s tests. To determine a cutoff value to distinguish different stages of TB infection Receiver Operating Characteristic (ROC) curve analysis using SPSS version 11.0 was used. All tests were calculated with a 0.05 of significance level and corrected using the Bonferroni method (0.0083).

#### Luminex HspX protein detection ELISA

Normality test using Shapiro-Wilk statistical analysis was p < 0.050. Further nonparametric Kruskal-Wallis One Way Analysis of Variance (ANOVA) on Ranks, with all pairwise multiple comparison procedures using Dunn's Method showed p < 0.05 for LTBI vs active TB groups, p<0.05 for control vs active groups, and p>0.05 for LTBI vs uninfected groups.

## Results

### Immunodetection of rHspX

To examine potential cross-reactivity with homologous proteins from related bacteria, whole cell lysates from strains listed in Materials and methods section were prepared and western blot analysis performed. Anti-HspX monoclonal antibodies IT-20 only recognized the recombinant HspX protein and HspX from *M*. *tuberculosis* strain H37Rv lysate (S3 and S4 Figs). The Luminex bead capture assay detected no HspX protein in stationary phase culture supernatants from *Mycobacterium* species possessing protein sequences similar to *M*. *tuberculosis*, including *M*. *rhodesiae*, *M*. *kansasii*, *M*. *fortuitum* and *M*. *vaccae*. Interestingly, no cross reactivity was detected in culture supernatants from *M*. *bovis* BCG. *M*. *tuberculosis* Beijing harvested in log phase did not produce detectable HspX.

### Detection of IgG and IgM antibodies against HspX protein in serum of patients by ELISA

**Fig 1:**
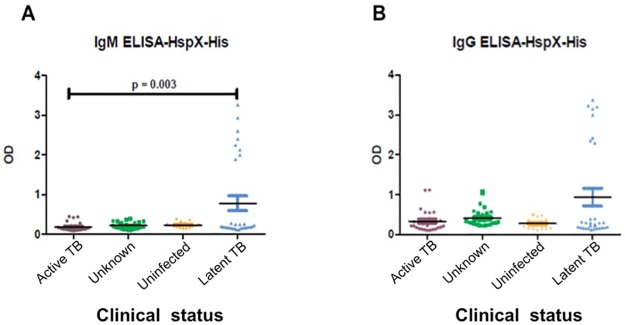
Detection of anti- HspX IgM and IgG in human sera by ELISA. Optical densities (492 nm) of IgM (A) or IgG antibodies (B) against recombinant HspX protein in individuals classified as active TB, unknown, uninfected and LTBI.

Anti-HspX IgG and IgM antibodies were detected in sera from patients classified as active TB, LTBI, unknown status, or uninfected individuals. For the antibody ELISA, levels of IgG and IgM antibodies were similar between non-infected and active TB patients. However, the level of reaction by the IgM antibodies was statistically higher (p = 0.003) in individuals classified with LTBI versus individuals with diagnosed active TB (Fig 1A). While IgG antibodies against HspX were detected in sera from all groups examined, there were no significant differences in level of reaction among the groups (Fig 1B).

**Fig 2:**
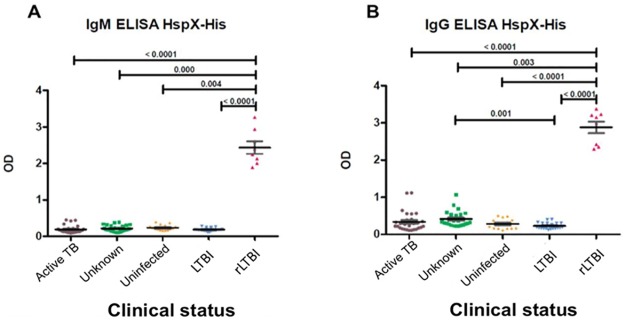
ELISA detection of anti-HspX IgM and IgG in serum of re-classified individuals. Optical densities (492 nm) of IgM (A) or IgG antibodies (B) against recombinant HspX in persons classified as active TB, unknown, uninfected, LTBI or rLTBI.

Separating recently infected LTBI (rLTBI) patients from all other LTBI patients according to Rabahi et al. [22], significant differences between IgM antibodies (Fig 2A) or IgG (Fig 2B) from individuals with rLTBI compared to all other groups was observed. ROC curve analysis based on OD_492_ values produced a cutoff point of 1.7 for IgG antibodies and a cutoff of 1.2 for IgM antibodies to identify an rLTBI patient.

### Detection of HspX protein in patient sera by ELISA

PBS and pooled normal human sera (NHS) were spiked with varying concentrations of rHspX. Using ECL chemiluminescent ELISA, detection limits of 10 pg/ml in PBS and 100 pg/ml in NHS were attained. Subsequently, serum samples were assessed from 5 human patients with recently diagnosed active TB disease and prior to initiation of anti-mycobacterial therapy, 5 TST-positive suspected LTBI cases, and 5 healthy TST-negative individuals. In this study, 4 of 5 human serum samples from individuals with active TB disease were positive while uninfected controls were below detectable levels. In the suspected LTBI group, 2 of 5 samples tested positive.

### Detection of HspX protein in patient sera by Luminex xMAP^®^ bead-capture ELISA

**Fig 3:**
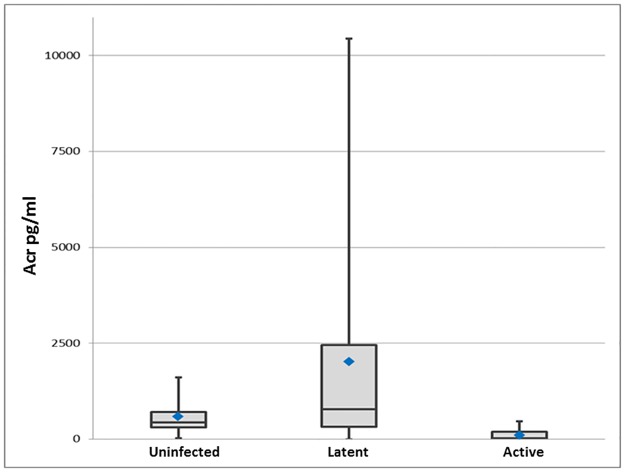
Scatter plot of HspX detection in clinical TB groups by Luminex bead-capture ELISA. Median for each group: uninfected 470 pg/ml, LTBI 860pg/ml and active TB 40 pg/ml. Normality test using Shapiro-Wilk statistical analysis was p < 0.050. Further nonparametric Kruskal-Wallis One Way Analysis of Variance (ANOVA) on Ranks, with all pairwise multiple comparison procedures using Dunn's Method showed p < 0.05 for LTBI versus active TB groups, p < 0.05 for uninfected versus active groups, and p > 0.05 for LTBI versus uninfected groups.

Twenty-four TST negative control sera were screened for HspX and a median level of 470 pg/ml was detected (Fig 3). Of the 23 putative samples from LTBI patients, 13 of 23 samples (56.5%) scored greater than 1 standard deviation above the background median with an average of 9,900pg/ml and a range of 1,000 to 36,000 pg/ml. Zero of 13 samples from patients with culture-confirmed active disease cases contained levels of HspX above the background median.

## Discussion

The goal of these studies was to determine the sensitivity and specificity of diagnostic approaches based upon the direct detection of antibodies against *M*. *tuberculosis* latency-associated alpha-crystallin (HspX) protein or upon the detection of the HspX protein itself in sera from individuals with suspected latent TB infection (LTBI).

Successful detection and treatment of individuals with LTBI constitutes a major impediment of worldwide TB control efforts. Although treatments for LTBI may exist, there currently are no specific diagnostics for the identification of cases in order to assess therapeutic efficacy; the lack of suitable biomarkers for mycobacterial latency is the major shortcoming of this effort. As a first step towards development of a LTBI diagnostic test based on targeting specific biomarkers, we present data examining detection of the *M*. *tuberculosis* HspX protein in patient sera. This secreted product belongs to the alpha crystallin superfamily of small heat shock stress proteins [23]. Investigators have referred to HspX as a largely restricted protein in individuals with LTBI [24]. In the present study, IgG and IgM antibodies produced against the HspX protein, and the protein itself were separately assayed in sera from confirmed active cases, suspected LTBI patients, and uninfected healthy controls.

Rabahi et al. [22] studied the humoral response against HspX and GlcB proteins from *M*. *tuberculosis* in health care workers, dividing population into three groups according to TST results: uninfected (negative PPD, previous LTBI (positive PPD) and recent LTBI (positive PPD conversion within one year). They found no differences in levels of IgG and IgM antibodies detected against both proteins in all three groups of individuals. However, the mean values for anti-HspX IgM were significantly higher among health care workers previously infected compared to uninfected controls, suggesting a possible role for this antigen in identifying recent LTBI patients. Our results showed similar ELISA values for anti-HspX IgG and IgM in sera from LTBI patients versus active TB, and in LTBI patients versus uninfected and unknown status groups. However, when we reclassified the groups to discriminate between active TB and LTBI, including recent LTBI (rLTBI) as suggested by Rabahiet al. [22], we found a significant difference in IgG and IgM levels between individuals with rLTBI and all the other groups. The ROC analysis shows sensitivity and a specificity of 100% with a cut off 1.7 for IgG and 1.2 for IgM (Fig 2). Interestingly, the recent-LTBI subgroup included only health care workers with continuous exposure to *M*. *tuberculosis* bacilli or its antigens. These results agree with the observations by Rabahi et al. [22] and support the idea of assaying for anti-HspX antibodies as a marker to identify recent LTBI.

Development of the antigen detection assay required the initial screen of several dozen anti-HspX monoclonal antibodies and the subsequent identification of the optimal capture and detection pair for use in the Luminex xMAP^®^bead-capture ELISA system. In our study, 13 of 23 TST-positive suspected LTBI cases (56.5%) scored one standard deviation above the median of the uninfected control population for the presence of serum HspX. The size of the error bar in Fig 3 and thus the variation in detected antigen level among the suspected LTBI patients was expected, confirming the hypothesis that LTBI is not a single stage of disease, but a spectrum, and demonstrating more clearly that the TST and IGRA tests cannot accurately diagnose it. These data provided support for the implementation of a second larger ongoing trial. Additionally, in the preliminary experiment, it was observed that 4 of 5 sera from individuals with culture-positive active infections contained measurable HspX. Those data contradict the results from this most recent study that show all 13 active cases have undetectable HspX serum levels. Interestingly, samples in the preliminary study were collected prior to the initiation of antimicrobial therapy while all 13 samples used in this Luminex study were from active disease patients with at least five months of standard therapy. Thus, the antigen detection assays also may be useful for assessing successful treatment regimens. Studies are being initiated to examine this potential.

A concern regarding the use of HspX as the target for these assays is the potential for cross reactivity with antigens from closely related species. It was hypothesized that antibodies against some antigenic sites on the *M*. *tuberculosis* HspX protein may share homology with similar proteins from species of environmental mycobacteria, and that all antigenic sites should cross-react with the sequence-identical proteins from *M*. *bovis* and *M*. *bovis* BCG [25]. To confirm this, immunoblot assays of sera from cattle infections showed reactivity to three antigens of 26, 22 and 16 kDa; however, the antibody reactivities to the 22 and 16 kDa proteins could be due to the tuberculin test, because reactivity was evident immediately following tuberculin testing of the animals [26]. In our studies, western blot analysis of cell lysates from different species of mycobacteria grown under laboratory oxygen depletion confirmed the published reports and did not show cross reactivity with the environmental mycobacteria, and did not cross react with *M*. *bovis*. Only HspX protein from *M*. *tu*berculosis H37Rv cell lysate was positive for the anti-HspX antibodies. It was expected that *M*. *tuberculosis* Beijing was recognized by anti-HspX antibodies. It is likely that the logarithmic phase growth conditions rather than the strain itself was responsible for the lack of HspX detection in our assays [6]. It was encouraging to note that no significant levels of cross reactivity were detected among the *M*. *tuberculosis*-complex species examined. Interestingly, as with the western blots, cross reactivity between *M*. *tuberculosis* and *M*. *bovis* (or BCG) HspX proteins were expected, but not detected. This finding could represent a difference in HspX expression and secretion pathways between *M*. *tuberculosis* and *M*. *bovis in vitro* versus *in vivo*, and is being actively investigated.

Regarding potential cross reactivity with the BCG vaccine, Geluk et al. [27] found that individuals infected with or exposed to *M*. *tuberculosis* responded well to HspX in the ELISPOT assay, while *M*. *tuberculosis*-unexposed individuals, including BCG-vaccinated individuals without any known exposure to *M*. *tuberculosis* had significantly lower responses to HspX. In the same paper, similar results were obtained with BCG-immunized mice, all suggesting that HspX expression by BCG after vaccination is low and unlikely to produce a significant immune response against HspX. Rabahi et al. [22] also reported no differences in IgM humoral responses to HspX in BCG-vaccinated and unvaccinated individuals. Slobbe et al. [28] demonstrated that BCG bacilli do not remain viable for more than three months post-vaccination, and it will not secrete HspX in the host. Thus, it is not expected that significant levels of anti-HspX antibodies or the HspX protein itself would be present in BCG-vaccinated individuals, and the likelihood of false positives is low. Nonetheless, ongoing studies will attempt to reduce potential sources of background by multiplexing the assays with additional *M*. *tuberculosis* complex-specific target antibodies and antigens such as for ESAT-6 and *M*. *tuberculosis*-specific cell wall glycans.

In conclusion, these tests have the potential to distinguish between patients that are truly infected with *M*. *tuberculosis* from those who have merely been exposed to non-tuberculous mycobacteria or have been vaccinated with BCG. It is proposed that both of these tests be run in tandem since the Luminex test would detect bacterial antigens rather than the host immune response to the bacillus, and should have the potential to identify *M*. *tuberculosis* infection in subjects with compromised immune systems, such as those with HIV/AIDS. Concomitantly, the ELISA test detecting the presence of antibody would be more sensitive and thus identify LTBI cases with lower numbers of infecting bacilli since there likely is a detection limit for the Luminex assay. The most exciting possibility is that both tests may identify individuals with LTBI who are at greatest risk of progressing to active disease and provide a metric for success in treating the identified infection. The assays would provide quantitative results that should reflect the burden of large numbers of actively-replicating bacteria, or few latent but viable bacteria capable of reactivating under proper conditions, in individuals with LTBI. Lastly, to enhance statistical confidence and determine if the assays can be used to measure therapeutic efficacy, we propose to collect and screen sera from a larger number of epidemiologically well-defined persons with a high likelihood of LTBI, persons unlikely to have LTBI, and persons with culture positive TB. In this study, we propose to obtain and screen subsequent monthly samples from individuals in these groups undergoing treatment for and LTBI.

## Supporting information

S1 Table Protein Blast search for *M*. *tuberculosis Rv2301c* gene. Organisms with homologous protein sequence.
(PDF)

S2 Table Percentage of similarity and divergence with HspX. (PDF)

S1 Fig Analysis of HspX protein in *M*. *tuberculosis* and other Mycobacterium species by western blot. (PDF)

S2 Fig Analysis of HspX protein in *M*. *tuberculosis* and other Mycobacterium species by western blot. (PDF)

## References

[1] World Health Organization Global Tuberculosis Report 20th edition. 2015. http://apps.who.int/iris/bitstream/10665/191102/1/9789241565059_eng.pdf

[2] Mack U, Migliori GB, Sester M, Rieder HL, Ehlers S, Goletti D, et al LTBI: latent tuberculosis infection or lasting immune responses to *M*. *tuberculosis*? A TBNET consensus statement. Eur Respir J. 2009; 33: 956–973. doi: 10.1183/09031936.00120908 19407047 10.1183/09031936.00120908

[3] World Health Organization. Guidelines on the management of latent tuberculosis infection. 2015 http://www.who.int/tb/publications/ltbi_document_page/en/25973515

[4] Gideon H, Flynn J. Latent tuberculosis: what the host “sees”? Immunol Res 2011; 50: 202–212. doi: 10.1007/s12026-011-8229-7 21717066 10.1007/s12026-011-8229-7 PMC3788603

[5] Institute of Medicine (US). Ending Neglect: The Elimination of Tuberculosis in the United States. Committee on the Elimination of Tuberculosis in the United States; Geiter L (ed), Washington, DC: National Academy Press; 2000. 25077271

[6] Hu Y, Coates A. Transcription of the stationary-phase associated *hspX* gene of *Mycobacterium tuberculosis* is inversely related to synthesis of the 16-kilodalton protein. J Bacteriol. 1999; 181: 1380–1387. 10049366 10.1128/jb.181.5.1380-1387.1999 PMC93524

[7] Haile Y, Bjune G, Wiker HG. Expression of the *mceA*, *esat-6* and *hspX* genes in *Mycobacterium tuberculosis* and their responses to aerobic conditions and to restricted oxygen supply. Microbiology. 2002; 148: 3881–3886. doi: 10.1099/00221287-148-12-3881 12480892 10.1099/00221287-148-12-3881

[8] Starck J, Källenius G, Marklund BI, Andersson DI, Akerlund T. Comparative proteome analysis of *Mycobacterium tuberculosis* grown under aerobic and anaerobic conditions. Microbiology. 2004; 150: 3821–3829. doi: 10.1099/mic.0.27284-0 15528667 10.1099/mic.0.27284-0

[9] Doherty TM. Separating latent and acute disease in the diagnosis of tuberculosis. In: Georgiev VS, Western KA, McGowan JJ, editors. New York, NY: National Institute of Allergy and Infectious Diseases, NIH, Humana Press; 2008 pp 91–99.

[10] Matty MA, Roca FJ, Cronan MR, Tobin DM. Adventures within the speckled band: heterogeneity, angiogenesis, and balanced inflammation in the tuberculous granuloma. Immunol Rev. 2015; 264: 276–287. doi: 10.1111/imr.12273 25703566 10.1111/imr.12273 PMC4704445

[11] Smith I. *Mycobacterium tuberculosis* pathogenesis and molecular determinants of virulence. Clin Microbiol Rev. 2003; 16: 463–496. doi: 10.1128/CMR.16.3.463-496.2003 12857778 10.1128/CMR.16.3.463-496.2003 PMC164219

[12] Gordillo S, Guirado E, Gi O, Díaz J, Amat I, Molinos S, et al Usefulness of acr expression for monitoring latent *Mycobacterium tuberculosis* bacilli in *'in vitro'* and *'in vivo'* experimental models. Scand J Immunol. 2006; 64: 30–39. doi: 10.1111/j.1365-3083.2006.01765.x 16784488 10.1111/j.1365-3083.2006.01765.x

[13] Sireci G, Dieli F, Di Liberto D, Buccheri S, La Manna MP, Scarpa F, et al Anti-16-kilodalton mycobacterial protein immunoglobulin m levels in healthy but purified protein derivative-reactive children decrease after chemoprophylaxis. Clin Vaccine Immunol. 2007; 14: 1231–1234. doi: 10.1128/CVI.00057-07 17626158 10.1128/CVI.00057-07 PMC2043321

[14] Limongi LCSA, Olival L, Conde MB, Junqueira-Kipnis AP. Determination of levels of specific IgA to the HspX recombinant antigen of *Mycobacterium tuberculosis* for the diagnosis of pleural tuberculosis. J Bras Pneumol. 2011; 37: 302–307. 21755184 10.1590/s1806-37132011000300005

[15] Raja A, Uma Devi KR, Ramalingam B, Brennan PJ. Immunoglobulin G, A, and M responses in serum and circulating immune complexes elicited by the 16-kilodalton antigen of *Mycobacterium tuberculosis*. Clin Diagn Lab Immunol. 2002; 9: 308–312. doi: 10.1128/CDLI.9.2.308-312.2002 11874868 10.1128/CDLI.9.2.308-312.2002 PMC119919

[16] Steingart KR, Henry M, Laal S, Hopewell PC, Ramsay A, Menzies D, et al Commercial serological antibody detection tests for the diagnosis of pulmonary tuberculosis: a systematic review. PLoS Med. 2007; 4: e202 doi: 10.1371/journal.pmed.0040202 17564490 10.1371/journal.pmed.0040202 PMC1891320

[17] Hamasur B, Bruchfeld J, van Helden P, Källenius G, Svenson SA. Sensitive urinary lipoarabinomannan test for tuberculosis. PLoS One. 2015; 10:e0123457 doi: 10.1371/journal.pone.0123457 25905641 10.1371/journal.pone.0123457 PMC4408114

[18] Qvist T, Johansen I, Pressler T, Høiby N, Andersen AB, Katzenstein TL, et al Urine lipoarabinomannan point-of-care testing in patients affected by pulmonary nontuberculous mycobacteria—experiences from the Danish Cystic Fibrosis cohort study. BMC Infect Dis. 2014; 14: 655 doi: 10.1186/s12879-014-0655-4 25471640 10.1186/s12879-014-0655-4 PMC4260379

[19] Quinn FD, Birkness KA, King PJ. Alpha-Crystallin as a potential marker of *Mycobacterium tuberculosis* latency. ASM News. 2002; 68: 612–617.

[20] Baker HN, Murphy R, Lopez E, Garcia C. Conversion of a capture ELISA to a Luminex xMAP assay using a multiplex antibody screening method. J Vis Exp. 2012; 65: e4084 doi: 10.3791/4084 22806215 10.3791/4084 PMC3471270

[21] Lin A, Nguyen L, Lee T, Clotilde LM, Kase JA, Son I, et al Rapid O serogroup identification of the ten most clinically relevant STECs by Luminex microbead-based suspension array. J Microbiol Methods. 2011; 87: 105–110. doi: 10.1016/j.mimet.2011.07.019 21835211 10.1016/j.mimet.2011.07.019

[22] Rabahi MF, Junqueira-Kipnis AP, Dos Reis MC, Oelemann W, Conde MB. Humoral response to HspX and GlcB to previous and recent infection by *Mycobacterium tuberculosis*. BMC Infect Dis. 2007; 7: 148 doi: 10.1186/1471-2334-7-148 18166139 10.1186/1471-2334-7-148 PMC2241823

[23] Kennaway CK, Benesch JL, Gohlke U, Wang L, Robinson CV, Orlova E V, et al Dodecameric structure of the small heat shock protein Acr1 from *Mycobacterium tuberculosis*. J Biol Chem. 2005; 280: 33419–33425. doi: 10.1074/jbc.M504263200 16046399 10.1074/jbc.M504263200

[24] Demissie A, Leyten EM, Abebe M, Wassie L, Aseffa A, Abate G, et al 2006. Recognition of stage-specific mycobacterial antigens differentiates between acute and latent infections with *Mycobacterium tuberculosis*. Clin Vaccine Immunol. 2006; 13: 179–186. doi: 10.1128/CVI.13.2.179-186.2006 16467323 10.1128/CVI.13.2.179-186.2006 PMC1391929

[25] Jiménez BA, Hinojoza-Loza E, Flores-Valdez MA, Prado-Montes de Oca E, Allen K, Estrada-Chávez C, et al 2013. Expression of non-replicating persistence associated genes of *Mycobacterium bovis* in lymph nodes from skin test-reactor cattle. Microb Pathog. 2013; 61–62: 23–28. doi: 10.1016/j.micpath.2013.04.012 23651670 10.1016/j.micpath.2013.04.012

[26] O'Loan CJ, Pollock JM, Hanna J, Neill SD. Immunoblot analysis of humoral immune responses to *Mycobacterium bovis* in experimentally infected cattle: early recognition of a 26-kilodalton antigen. Clin Diagn Lab Immunol. 1994; 1: 608–611. 8556509 10.1128/cdli.1.5.608-611.1994 PMC368349

[27] Geluk A, Lin MY, van Meijgaarden KE, Leyten EM, Franken KL, Ottenhoff TH, et al T-Cell Recognition of the HspX protein of *Mycobacterium tuberculosis* correlates with latent *M*. *tuberculosis* infection but not with *M*. *bovis* BCG vaccination. Infect Immun. 2007; 75: 2914–2921. doi: 10.1128/IAI.01990-06 17387166 10.1128/IAI.01990-06 PMC1932904

[28] Slobbe L, Lockhart E, O'Donnell MA, MacKintosh C, De Lisle G, Buchan G. An *in vivo* comparison of bacillus Calmette-Guérin (BCG) and cytokine-secreting BCG vaccines. Immunol. 1999; 96: 517–523. 10.1046/j.1365-2567.1999.00702.x PMC2326794 10233736

