# Supplementary material for: Detection of anti-HspX antibodies and HspX protein in patient sera for the identification of recent latent infection by Mycobacterium tuberculosis
Source: PLoS One. 2017 Aug 16;12(8):e0181714. doi: 10.1371/journal.pone.0181714 (PMC5558980; doi:10.1371/journal.pone.0181714)
Supplement: S1 Table — Organisms with homologous protein sequence. (PDF) [file pone.0181714.s001.pdf]

S1 Table. Protein Blast search for *M. tuberculosis Rv2301c* gene. Organisms with homologous protein sequence.		
**1**	**NP_216547.1**	HspX gene product [*Mycobacterium tuberculosis* H37Rv]
**2**	**NP_855707.1**	Heat shock protein hspX [*Mycobacterium bovis* AF2122/97]
**3**	**YP_978140.1**	Heat shock protein hspX [*Mycobacterium bovis* BCG str. Pasteur 1173P2]
**4**	**YP_003031913.1**	Heat shock protein hspX [*Mycobacterium tuberculosis* KZN 1435]
**5**	**ZP_04749296.1**	Heat shock protein [*Mycobacterium kansasii* ATCC 12478]
**6**	**YP_001851758.1**	Heat shock protein HspX [*Mycobacterium marinum* M]
**7**	**ZP_06852440.1**	Heat shock protein hspX [Mycobacterium parascrofulaceum ATCC BAA-614]
**8**	**ZP_04750177.1**	Heat shock protein [*Mycobacterium kansasii* ATCC 12478]
**9**	**YP_888221.1**	Hypothetical protein [*Mycobacterium smegmatis* str. MC2 155]
**10**	**YP_954724.1**	Heat shock protein Hsp20 [*Mycobacterium vanbaalenii* PYR-1]
**11**	**NP_962635.1**	Hsp [*Mycobacterium avium subsp. paratuberculosis* K-10]
**12**	**ZP_05218861.1**	Hsp20/alpha crystallin family protein *[Mycobacterium avium subsp. avium* ATCC 25291]
**13**	**ZP_05225418.1**	Hsp20/alpha crystallin family protein [*Mycobacterium intracellulare* ATCC 13950]
**14**	**NP_000385.1**	Alpha-crystallin A chain [*Homo sapiens*]
**15**	**NP_001876.1**	Alpha-crystallin B chain [*Homo sapiens*]
**16**	**NP_653218.1**	Heat shock protein beta-6 [*Homo sapiens*]
**17**	**YP_002503848.1**	Hypothetical protein MLBr_01795 [*Mycobacterium leprae* Br4923]
**18**	**Q57733.1**	RecName: Full=Small heat shock protein HSP16.5
**19**	**AAB85357.1**	Heat shock protein, class I [*Methanothermobacter thermautotrophicus* str. Delta H]
**20**	**NP_143673.1**	Hypothetical protein PH1842 [*Pyrococcus horikoshii* OT3]
**21**	**NP_148279.2**	Small heat shock protein [*Aeropyrum pernix* K1]
**22**	**NP_070125.1**	Heat shock protein 20 [*Archaeoglobus fulgidus* DSM 4304]
**23**	**NP_070795.1**	Heat shock protein 20 [*Archaeoglobus fulgidus* DSM 4304]
**24**	**CAC11613.1**	Small heat shock protein (hsp20) related protein [*Thermoplasma acidophilum*]
**25**	**CAC11993.1**	Small heat shock protein (hsp20) related protein [*Thermoplasma acidophilum*]
**26**	**AAG20020.1**	Small heat shock protein [*Halobacterium sp*. NRC-1]
**27**	**AAG20865.1**	Heat shock protease protein [*Halobacterium sp.* NRC-1]
**28**	**NP_213880.1**	Small heat shock protein (class I) [*Aquifex aeolicus* VF5]
**29**	**BAB13269.1**	16 kDa heat shock protein A [*Buchnera aphidicola* str. APS (*Acyrthosiphon pisum*)]
**30**	**CAA46374.1**	Heat shock protein [*Clostridium acetobutylicum* ATCC 824]
**31**	**P12809.1**	RecName: Full=18 kDa antigen; AltName: Full=HSP 16.7
**32**	**AAG06514.1**	Heat-shock protein IbpA [*Pseudomonas aeruginosa* PAO1]
**33**	**CAA14735.1**	Heat shock protein (hsp22) [*Rickettsia prowazekii* str. Madrid E]
**34**	**BAA16996.1**	16.6 kDa small heat shock protein, molecular chaperon [*Synechocystis sp*. PCC 6803]
**35**	**NP_228185.1**	Heat shock protein, class I [*Thermotoga maritima* MSB8]
**36**	**AAF93196.1**	16 kDa heat shock protein A [*Vibrio cholerae* O1 biovar El Tor str. N16961]
**37**	**AAF85033.1**	Low molecular weight heat shock protein [*Xylella fastidiosa* 9a5c]
**38**	**P96193.1**	RecName: Full=16 kDa heat shock protein B
**39**	**S49042**	Global stress protein gspA - *Legionella pneumophila*
**40**	**CAA67831.1**	Heat shock protein [*Oenococcus oeni*]
**41**	**CAB99442.1**	HspA protein [*Stigmatella aurantiaca* DW4/3-1]
**42**	**Q53595.1**	RecName: Full=18 kDa heat shock protein; AltName: Full=HSP 18
**43**	**BAA32501.1**	HspA [*Thermosynechococcus vulcanus*]
