# Supplementary material for: Detection of anti-HspX antibodies and HspX protein in patient sera for the identification of recent latent infection by Mycobacterium tuberculosis
Source: PLoS One. 2017 Aug 16;12(8):e0181714. doi: 10.1371/journal.pone.0181714 (PMC5558980; doi:10.1371/journal.pone.0181714)
Supplement: S2 Table — (PDF) [file pone.0181714.s002.pdf]

S2 Table. Percentage of identity, DNAstar analysis

*Sequence Distances of Untitled ClustalW (Slow/Accurate, Gonnet)
*Upper triangle shows identity percentage
*Lower triangle shows divergence percentage

Percent Identity

	1	2	3	4	5	6	7	8	9	10	11	12	13	14	15	16	17	18	19	20	21	22	23	24	25	26	27	28	29	30	31	32	33	34	35	36	37	38	39	40	41	42	43		
1		100.0	100.0	100.0	80.4	71.3	71.3	72.7	60.8	49.7	32.6	32.6	31.9	16.1	15.9	14.8	26.4	19.8	19.4	20.9	18.3	26.2	31.0	7.8	24.4	17.3	24.8	24.6	18.1	20.3	26.4	16.4	18.8	25.0	24.3	18.5	27.0	14.3	20.6	25.5	24.3	27.3	24.5	1	NP_216547.1
2	0.0		100.0	100.0	80.4	71.3	71.3	72.7	60.8	49.7	32.6	32.6	31.9	16.1	15.9	14.8	26.4	19.8	19.4	20.9	18.3	26.2	31.0	7.8	24.4	17.3	24.8	24.6	18.1	20.3	26.4	16.4	18.8	25.0	24.3	18.5	27.0	14.3	20.6	25.5	24.3	27.3	24.5	2	NP_855707.1
3	0.0	0.0		100.0	80.4	71.3	71.3	72.7	60.8	49.7	32.6	32.6	31.9	16.1	15.9	14.8	26.4	19.8	19.4	20.9	18.3	26.2	31.0	7.8	24.4	17.3	24.8	24.6	18.1	20.3	26.4	16.4	18.8	25.0	24.3	18.5	27.0	14.3	20.6	25.5	24.3	27.3	24.5	3	YP_978140.1
4	0.0	0.0	0.0		80.4	71.3	71.3	72.7	60.8	49.7	32.6	32.6	31.9	15.8	15.6	13.7	26.2	19.7	19.3	19.9	17.8	25.6	31.0	7.6	24.4	16.4	24.8	24.6	18.0	19.3	26.2	16.4	17.4	24.8	23.9	18.5	26.4	14.3	20.6	24.6	23.2	27.1	24.3	4	YP_003031913.1
5	22.7	22.7	22.7	22.7		67.6	76.9	66.9	64.8	48.6	28.4	28.4	27.7	16.2	16.1	13.4	27.4	19.8	19.4	20.1	19.8	25.4	30.2	9.2	23.6	16.5	28.1	25.4	19.0	18.8	27.4	17.3	17.4	25.0	30.4	19.4	29.3	16.7	23.0	25.5	25.2	28.3	25.2	5	ZP_04749296.1
6	36.1	36.1	36.1	36.1	42.3		62.7	72.0	62.2	49.0	30.5	30.5	31.9	20.6	17.5	15.7	26.6	23.1	21.8	20.3	24.0	24.8	28.7	10.7	26.8	16.7	28.9	27.8	16.1	22.6	26.6	16.4	21.2	26.6	26.7	18.5	30.0	16.5	21.3	24.3	26.6	29.9	28.3	6	YP_001851758.1
7	36.1	36.1	36.1	36.1	27.6	51.3		65.5	62.7	47.2	29.1	29.1	28.4	16.9	15.3	13.4	21.8	18.3	17.9	22.4	19.8	24.6	28.7	11.3	24.4	18.0	26.4	23.1	15.3	20.3	21.8	15.0	16.7	23.6	25.2	16.3	27.9	15.9	18.5	27.0	25.2	23.6	25.2	7	ZP_06852440.1
8	33.9	33.9	33.9	33.9	43.5	35.0	46.0		58.7	48.3	34.0	34.0	34.8	20.6	20.4	19.7	29.0	21.5	16.5	20.3	20.8	25.6	33.3	10.7	27.6	18.8	24.0	28.6	15.3	21.9	29.0	17.2	22.6	21.6	25.2	21.5	27.9	16.5	20.6	24.3	26.6	27.6	24.6	8	ZP_04750177.1
9	54.9	54.9	54.9	54.9	47.3	52.1	51.3	59.2		47.9	29.8	29.8	31.2	17.4	12.3	20.3	23.8	17.6	20.1	22.4	22.2	23.8	28.5	12.1	29.3	17.4	25.4	22.4	16.5	20.4	23.8	22.1	19.6	26.6	27.2	23.5	29.1	18.5	21.0	27.2	25.0	25.6	27.5	9	YP_888221.1
10	80.8	80.8	80.8	80.8	83.7	82.7	87.7	84.6	85.6		28.4	28.4	27.7	16.7	12.2	17.9	20.7	19.2	20.3	21.8	19.2	21.6	24.0	13.6	22.0	18.1	26.4	29.3	11.9	23.4	20.7	18.6	19.0	25.2	25.2	17.6	23.5	17.4	15.3	25.7	28.4	27.6	26.8	10	YP_954724.1
11	144.6	144.6	144.6	144.6	170.9	156.9	166.0	137.3	161.3	170.9		99.3	87.7	19.1	19.7	17.3	22.2	20.0	18.8	22.5	13.5	21.4	23.9	10.3	24.2	19.0	27.9	18.8	12.2	20.7	22.2	24.4	21.2	25.2	28.3	22.7	28.7	19.4	17.5	22.1	28.2	23.8	23.9	11	NP_962635.1
12	144.6	144.6	144.6	144.6	170.9	156.9	166.0	137.3	161.3	170.9	0.7		87.0	19.9	19.0	16.5	21.4	19.3	18.1	21.7	12.7	20.6	23.1	10.3	24.2	18.3	27.9	18.1	12.2	20.0	21.4	24.4	21.2	25.2	27.5	22.7	28.0	19.4	17.5	22.1	27.5	23.1	23.9	12	ZP_05218861.1
13	148.5	148.5	148.5	148.5	176.1	148.5	170.9	133.8	152.6	176.1	13.5	14.3		21.3	21.9	18.1	20.6	20.0	17.4	25.4	14.3	20.6	25.4	11.7	24.2	19.0	26.2	19.6	12.2	20.0	20.6	23.7	19.0	25.2	28.3	22.0	29.4	19.4	17.5	21.4	25.4	24.6	23.9	13	ZP_05225418.1
14	307.0	307.0	307.0	313.0	305.0	239.0	291.0	239.0	284.0	296.0	257.0	248.0	230.0		57.7	45.4	16.0	17.7	18.0	17.0	16.7	12.1	18.9	9.8	16.0	14.1	25.0	21.2	14.1	13.6	16.0	14.7	17.6	14.6	21.3	13.6	20.9	16.4	16.2	14.6	20.3	13.6	15.3	14	NP_000385.1
15	309.0	309.0	309.0	315.0	307.0	281.0	321.0	240.0	403.0	407.0	249.0	260.0	224.0	61.3		50.0	15.7	16.0	13.5	16.6	15.6	13.2	15.7	11.2	18.2	14.6	21.5	15.9	12.5	14.8	15.7	14.5	20.3	11.6	18.2	16.4	17.9	14.0	15.2	15.2	18.1	14.1	10.2	15	NP_001876.1
16	332.0	332.0	332.0	362.0	371.0	313.0	371.0	250.0	241.0	274.0	284.0	298.0	271.0	93.1	79.9		15.6	15.4	15.7	17.0	16.4	13.1	22.3	9.6	15.8	17.0	19.1	20.6	10.5	13.6	15.6	13.3	16.7	10.9	16.2	16.1	18.4	15.9	12.8	11.6	19.2	17.7	15.6	16	NP_653218.1
17	186.0	186.0	186.0	187.8	177.9	184.3	226.0	166.3	205.0	237.0	220.0	229.0	238.0	307.0	315.0	317.0		20.9	21.7	23.4	22.8	20.9	30.6	11.6	22.4	19.4	21.6	32.8	16.3	25.4	100.0	14.6	14.6	26.8	31.1	16.4	22.3	13.2	12.7	19.5	25.0	55.4	25.4	17	YP_002503848.1
18	248.0	248.0	248.0	250.0	248.0	212.0	268.0	228.0	281.0	255.0	246.0	255.0	246.0	279.0	307.0	319.0	234.0		30.0	35.6	29.5	39.2	25.9	21.8	21.1	19.7	20.8	29.5	14.6	21.8	20.9	14.3	19.3	19.3	25.2	18.3	18.1	11.3	14.8	21.8	26.1	25.6	23.1	18	Q57733.1
19	253.0	253.0	253.0	255.0	253.0	224.0	274.0	298.0	244.0	241.0	261.0	271.0	284.0	273.0	365.0	313.0	226.0	159.9		32.9	28.3	31.5	25.9	17.9	21.1	20.7	20.8	36.8	13.7	24.4	21.7	14.7	15.3	22.5	31.0	16.5	21.3	15.6	12.3	25.0	30.7	24.8	26.3	19	AAB85357.1
20	236.0	236.0	236.0	248.0	244.0	241.0	219.0	241.0	219.0	224.0	218.0	226.0	195.0	289.0	298.0	289.0	209.0	129.7	143.3		37.5	39.0	32.8	18.8	26.2	19.0	27.3	40.1	10.6	25.4	23.4	12.4	16.8	21.0	33.1	11.9	23.1	11.0	10.7	22.9	24.5	28.0	27.7	20	NP_143673.1
21	270.0	270.0	270.0	276.0	248.0	204.0	248.0	237.0	220.0	257.0	368.0	393.0	347.0	296.0	315.0	299.0	214.0	163.5	171.0	121.4		45.9	32.8	20.0	24.1	19.6	25.4	32.5	15.9	20.0	22.8	16.4	18.8	16.7	31.5	16.5	20.8	15.7	14.5	18.3	22.0	22.4	24.0	21	NP_148279.2
22	187.8	187.8	187.8	193.1	194.7	197.0	198.0	192.9	205.0	228.0	229.0	238.0	238.0	410.0	377.0	380.0	236.0	114.4	150.9	115.2	91.6		36.1	22.7	26.5	22.9	29.6	36.5	13.4	23.8	20.9	16.4	15.8	15.9	31.2	19.0	22.3	12.4	14.4	19.0	25.2	20.5	20.8	22	NP_070125.1
23	153.8	153.8	153.8	153.8	158.5	168.7	168.7	140.9	170.2	203.0	205.0	211.0	194.9	261.0	313.0	219.0	155.9	190.0	190.3	143.4	143.8	127.7		17.9	23.0	20.7	22.7	38.1	15.0	24.0	30.6	20.5	15.9	24.2	32.4	18.5	31.9	20.5	16.4	24.6	26.7	30.5	28.2	23	NP_070795.1
24	736.0	736.0	736.0	796.0	569.0	471.0	442.0	471.0	414.0	365.0	493.0	493.0	426.0	531.0	451.0	538.0	430.0	224.0	274.0	261.0	246.0	216.0	276.0		13.7	14.7	16.4	17.4	15.9	17.1	11.6	12.9	10.8	9.0	18.1	14.6	10.8	12.9	12.7	12.2	9.6	12.8	17.5	24	CAC11613.1
25	200.0	200.0	200.0	200.0	208.0	182.5	200.0	176.2	164.7	223.0	202.0	202.0	202.0	309.0	271.0	313.0	218.0	232.0	232.0	187.4	202.0	184.8	213.0	360.0		19.5	23.3	26.0	15.6	22.3	22.4	16.7	23.8	18.7	31.5	19.5	17.1	14.2	14.8	28.1	18.9	23.5	23.8	25	CAC11993.1
26	285.0	285.0	285.0	299.0	298.0	296.0	274.0	261.0	284.0	271.0	258.0	268.0	258.0	352.0	339.0	289.0	254.0	249.0	237.0	260.0	252.0	214.0	237.0	337.0	252.0		22.7	22.3	10.6	19.7	19.4	13.3	12.8	18.2	22.5	17.6	19.2	14.9	13.1	16.7	21.4	19.5	21.1	26	AAG20020.1
27	197.0	197.0	197.0	197.0	172.8	167.0	185.6	204.0	194.6	185.6	174.5	174.5	187.4	195.0	229.0	258.0	228.0	236.0	236.0	179.0	194.4	162.7	216.0	301.0	210.0	216.0		26.7	14.9	16.1	21.6	22.7	21.8	24.2	28.1	17.9	28.9	19.3	16.5	25.6	20.2	21.0	24.4	27	AAG20865.1
28	198.0	198.0	198.0	198.0	194.9	174.9	211.0	169.5	219.0	164.3	261.0	271.0	252.0	231.0	309.0	238.0	143.6	163.2	124.4	110.9	145.1	125.7	118.8	284.0	189.3	219.0	183.8		14.5	26.1	32.8	17.6	16.2	27.7	39.7	25.6	27.9	15.6	17.4	25.9	33.1	31.1	36.0	28	NP_213880.1
29	271.0	271.0	271.0	274.0	260.0	307.0	321.0	321.0	298.0	422.0	407.0	407.0	407.0	352.0	399.0	482.0	303.0	339.0	362.0	482.0	311.0	371.0	328.0	311.0	317.0	482.0	332.0	342.0		13.9	16.3	28.1	12.9	21.4	19.7	22.5	15.3	25.7	27.7	17.6	20.5	13.8	21.6	29	BAB13269.1
30	243.0	243.0	243.0	254.0	261.0	216.0	243.0	224.0	240.0	209.0	237.0	246.0	246.0	365.0	335.0	362.0	194.7	224.0	200.0	195.1	246.0	205.0	203.0	287.0	219.0	249.0	305.0	188.4	357.0		25.4	19.8	17.6	20.4	21.0	15.7	25.2	15.4	15.7	34.9	22.4	22.3	20.6	30	CAA46374.1
31	186.0	186.0	186.0	187.8	177.9	184.3	226.0	166.3	205.0	237.0	220.0	229.0	238.0	307.0	315.0	317.0	0.0	234.0	226.0	209.0	214.0	236.0	155.9	430.0	218.0	254.0	228.0	143.6	303.0	194.7		14.6	14.6	26.8	31.1	16.4	22.3	13.2	12.7	19.5	25.0	55.4	25.4	31	P12809.1
32	299.0	299.0	299.0	299.0	285.0	299.0	328.0	287.0	222.0	265.0	200.0	200.0	206.0	335.0	342.0	374.0	337.0	347.0	335.0	399.0	301.0	301.0	240.0	386.0	296.0	371.0	216.0	279.0	172.9	248.0	337.0		19.4	20.1	23.4	41.4	18.1	74.8	53.4	19.2	17.1	15.0	16.5	32	AAG06514.1
33	261.0	261.0	261.0	282.0	284.0	232.0	296.0	216.0	252.0	260.0	232.0	232.0	260.0	281.0	243.0	296.0	337.0	255.0	321.0	294.0	261.0	311.0	309.0	466.0	206.0	386.0	224.0	305.0	383.0	279.0	337.0	253.0		16.7	19.7	22.9	14.9	18.0	19.1	22.7	21.8	12.7	19.7	33	CAA14735.1
34	195.0	195.0	195.0	196.0	195.0	184.2	208.0	228.0	184.2	196.7	196.8	196.8	196.8	339.0	434.0	466.0	183.0	255.0	218.0	233.0	296.0	311.0	201.0	586.0	264.0	271.0	202.0	175.5	229.0	240.0	183.0	244.0	296.0		23.7	14.6	22.9	17.3	20.6	29.6	30.3	28.2	56.6	34	BAA16996.1
35	201.0	201.0	201.0	204.0	157.6	183.8	196.7	196.7	179.5	196.7	171.7	177.0	171.7	230.0	270.0	305.0	153.5	196.7	153.9	142.1	150.9	152.3	146.1	273.0	151.2	217.0	172.8	112.6	249.0	233.0	153.5	209.0	249.0	206.0		24.8	25.7	20.6	20.7	26.5	30.0	30.9	31.9	35	NP_228185.1
36	267.0	267.0	267.0	267.0	254.0	267.0	303.0	228.0	209.0	279.0	215.0	215.0	223.0	362.0	299.0	305.0	299.0	268.0	298.0	418.0	298.0	258.0	267.0	339.0	253.0	281.0	274.0	193.2	217.0	313.0	299.0	106.4	213.0	337.0	196.0		19.3	38.2	41.7	17.3	19.9	18.5	14.7	36	AAF93196.1
37	181.5	181.5	181.5	186.1	164.6	159.9	174.6	174.6	166.0	209.0	168.8	173.8	164.0	234.0	276.0	267.0	219.0	271.0	231.0	212.0	237.0	219.0	148.9	466.0	289.0	257.0	167.0	174.6	321.0	196.8	219.0	273.0	332.0	213.0	192.1	255.0		19.0	16.8	21.1	27.7	24.8	30.1	37	AAF85033.1
38	347.0	347.0	347.0	347.0	296.0	298.0	309.0	298.0	265.0	284.0	253.0	253.0	253.0	299.0	354.0	311.0	377.0	447.0	317.0	456.0	313.0	403.0	240.0	383.0	349.0	330.0	254.0	317.0	192.1	321.0	377.0	30.7	273.0	285.0	239.0	118.6	258.0		48.6	14.7	18.1	12.9	18.2	38	P96193.1
39	239.0	239.0	239.0	239.0	213.0	230.0	265.0	239.0	233.0	323.0	281.0	281.0	281.0	305.0	325.0	389.0	393.0	332.0	403.0	471.0	339.0	344.0	299.0	393.0	335.0	377.0	298.0	284.0	175.7	315.0	393.0	71.3	257.0	239.0	237.0	105.4	294.0	83.6		19.7	17.9	12.4	18.5	39	S49042
40	193.4	193.4	193.4	198.0	193.4	201.0	181.1	201.0	179.5	191.7	221.0	221.0	229.0	339.0	323.0	430.0	252.0	224.0	195.0	214.0	270.0	258.0	198.0	407.0	172.8	296.0	192.5	190.0	279.0	132.9	252.0	255.0	216.0	162.7	185.4	284.0	232.0	335.0	250.0		26.2	20.5	29.1	40	CAA67831.1
41	201.0	201.0	201.0	210.0	196.7	184.2	196.7	184.2	195.0	170.8	172.3	177.5	195.1	243.0	273.0	257.0	195.0	188.4	155.9	200.0	222.0	196.6	183.4	545.0	261.0	229.0	244.0	142.2	239.0	218.0	195.0	287.0	226.0	157.8	159.9	248.0	175.4	271.0	274.0	187.6		27.5	33.1	41	CAB99442.1
42	178.5	178.5	178.5	180.1	171.0	160.4	207.0	176.8	193.1	176.4	205.0	212.0	198.0	362.0	352.0	277.0	66.5	193.2	196.0	173.3	218.0	239.0	157.0	389.0	208.0	252.0	233.0	153.5	360.0	219.0	66.5	328.0	393.0	171.8	154.5	267.0	196.0	386.0	399.0	240.0	177.0		27.7	42	Q53595.1
43	200.0	200.0	200.0	201.0	196.7	171.7	196.7	198.0	177.0	182.6	204.0	204.0	204.0	321.0	498.0	315.0	194.7	211.0	187.0	175.5	204.0	237.0	171.8	282.0	206.0	232.0	200.0	127.9	228.0	239.0	194.7	298.0	249.0	63.9	148.7	335.0	159.5	271.0	265.0	166.0	142.1	175.8		43	BAA32501.1
	1	2	3	4	5	6	7	8	9	10	11	12	13	14	15	16	17	18	19	20	21	22	23	24	25	26	27	28	29	30	31	32	33	34	35	36	37	38	39	40	41	42	43		
