# Supplementary figures and images for: Detection of anti-HspX antibodies and HspX protein in patient sera for the identification of recent latent infection by Mycobacterium tuberculosis
Source: PLoS One. 2017 Aug 16;12(8):e0181714. doi: 10.1371/journal.pone.0181714 (PMC5558980; doi:10.1371/journal.pone.0181714)

S1 Fig

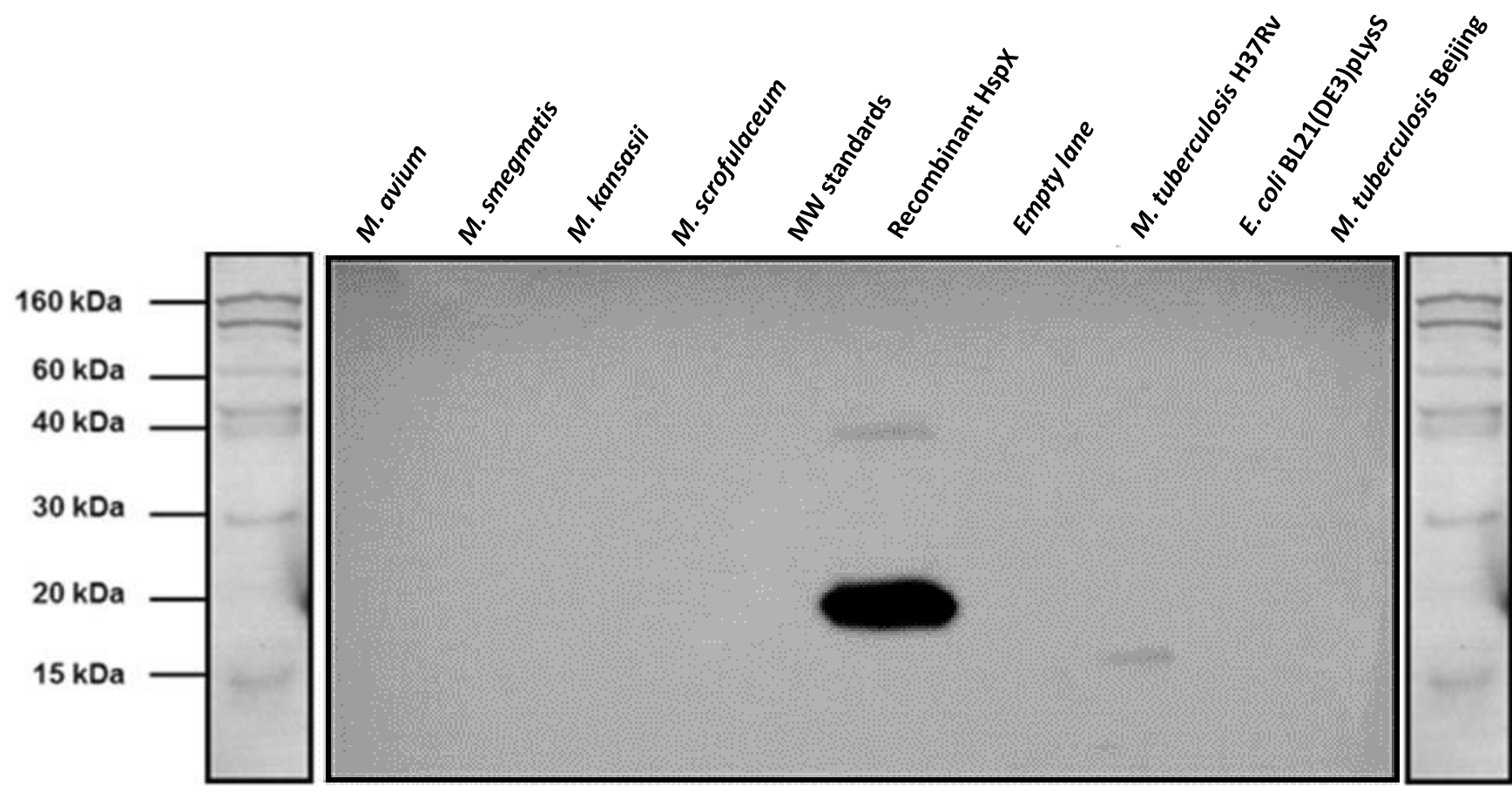

Supplement: S1 Fig — (PDF) [file pone.0181714.s003.pdf]

S2 Fig

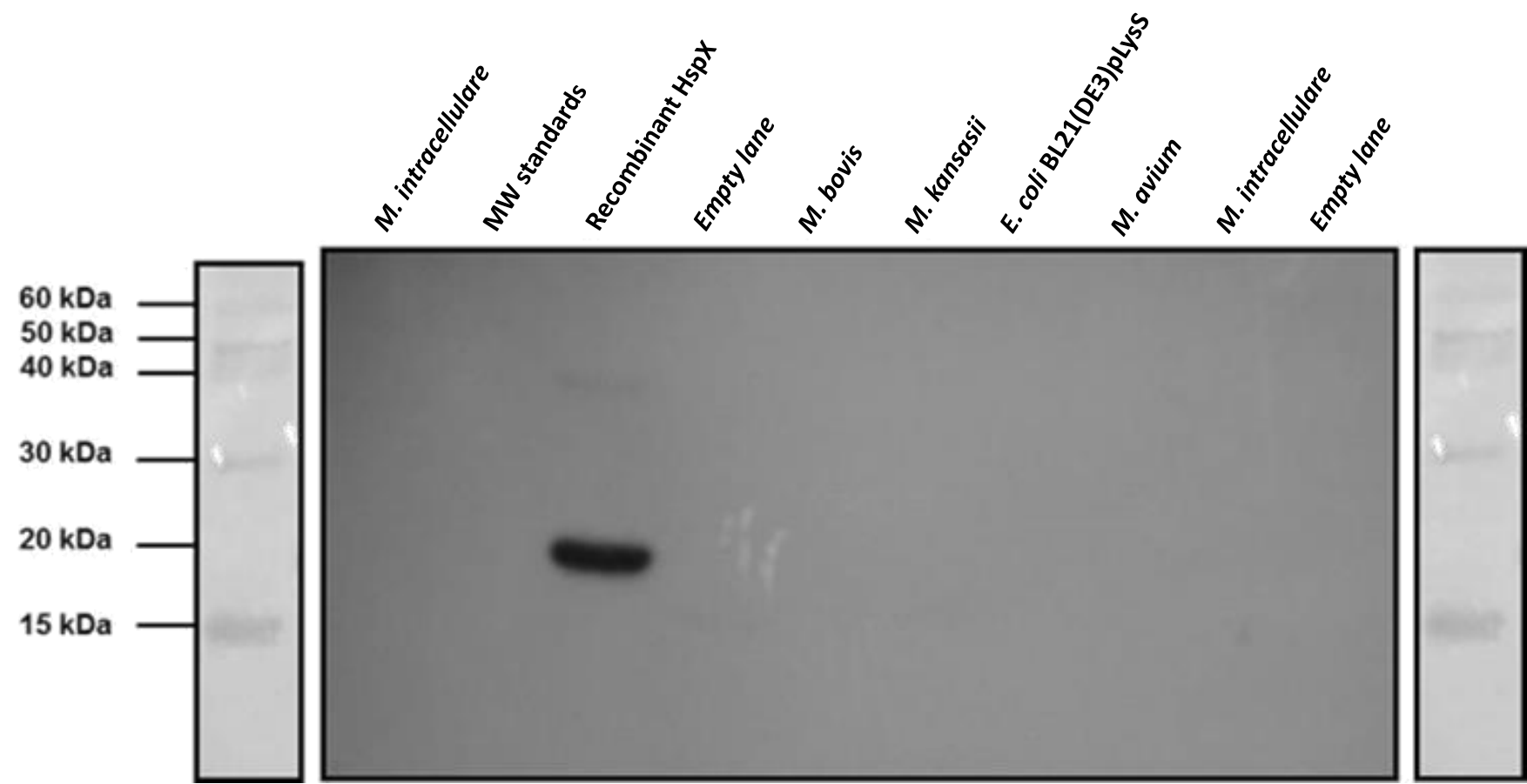

Supplement: S2 Fig — (PDF) [file pone.0181714.s004.pdf]
